# Link between metacognition and social cognition in schizophrenia: a systematic review and meta-analysis

**DOI:** 10.3389/fpsyt.2023.1285993

**Published:** 2023-12-22

**Authors:** Alex Motut, Clémence Isaac, Marie-Carmen Castillo, Dominique Januel

**Affiliations:** ^1^Centre de Recherche Clinique 93G03, Etablissement Public de Santé de Ville-Evrard, Neuilly-sur-Marne, France; ^2^Laboratoire Psychopathologie et Processus de Changement, Université Paris 8, Saint-Denis, France

**Keywords:** metacognition, social cognition, schizophrenia, cognition, mental health

## Abstract

**Introduction:**

Metacognition is the ability to reflect on one’s own cognitive processes, monitor and regulate them to enhance mental performance. Social cognition involves the capacity to perceive and respond to social cues from others. The study of metacognition and social cognition is an expanding research field in psychiatry. Both domains are related to neurocognition, symptoms and psychosocial functioning in schizophrenia. Understanding the relationship between social cognition and metacognition may be pivotal for enhancing the treatment of cognitive symptoms in schizophrenia.

**Methods:**

We conducted a PRISMA systematic review and meta-analysis on quantitative studies comparing metacognition to social cognitive outcomes in adult outpatients with a schizophrenia spectrum disorder. Reports were retrieved from the Medline, ScienceDirect and PsycINFO databases up to July 13th, 2023. Risk of bias was assessed with the Cochrane tool.

**Results:**

Our review included 1,036 participants across 17 reports, with 12 reports included in the meta-analysis. We found a significant positive correlation (*r* = 0.28, 95% CI: [0.14, 0.41]) between social cognition and metacognition. Subgroup analyses indicated that metacognition was specifically associated with theory of mind, attribution, and emotion processing. Different patterns of correlations were observed according to the assessment of metacognition and its subdimensions.

**Conclusion:**

Despite discrepancies among the included studies, no publication bias was detected. The results suggest that metacognition and social cognition are distinct but related constructs. Those processes should be assessed and treated together, along with neurocognition, in schizophrenia.

## Introduction

During the last 30 years, metacognition has become one of the main areas of cognitive research. Originally, metacognition was defined by Flavell as knowledge of one’s own cognitive processes, their products, and everything related to them (1). This concept is used to describe not only reflection upon specific mental experiences (i.e., thoughts or sensations) but also a more synthetic process of integrating thoughts, intentions, emotions and relationships over events to form a dynamic representation of the self and others (2).

This definition was expanded to include three distinct functional components. First, metacognitive knowledge refers to acquired knowledge about cognitive processes (e.g., knowing that one is more attentive in a quiet place than in a noisy place). Second, metacognitive monitoring corresponds to the assessment of cognitive functioning (e.g., evaluating one’s understanding of a read text). Finally, metacognitive regulation or metacognitive control refer to the reorientation of cognitive activity toward cognitive performance (e.g., using a new strategy to memorize a complex text). Monitoring and regulation thus operate between two separate levels: a cognitive “object-level” and a “meta-level” (3). These components are conceptualized as metacognitive awareness. Overall, metacognition is a useful concept for cognitive activities as it increases the effectiveness and efficiency of cognitive functions (4, 5).

Social cognition is generally defined as the set of mental operations underlying social interactions. These mental operations make it possible to infer intentions and produce behaviors (6). It is composed of theory of mind, emotion processing, attributional style, social perception, and social knowledge (6). Theory of mind is the ability to identify the thoughts and mental states of others. It includes three orders (i.e., level of representation) and two processes: cognitive (i.e., understanding others’ thoughts regardless of emotions) and emotional (i.e., inferring others’ emotional mental states) (7). Emotion processing is the ability to perceive and use emotions. Attribution style is the way in which individuals explain the causes of events. Social perception represents how individuals perceive social cues and social knowledge refers to the awareness of the functioning of society and social interactions (6). These social cognitive functions support the roles, rules and goals of social interactions (8).

Metacognition is a rapidly expanding field of study in psychiatric disorders (i.e., schizophrenia, mood disorders, substance-related disorders, anxiety) because metacognitive deficits seem to be a common feature of psychiatric disorders, particularly psychosis (9, 10). The most impaired metacognitive process in schizophrenia may be mastery (11–13), which is the ability to work with one’s mental representations and states, in order to implement effective action strategies for performing cognitive tasks or coping with problematic mental states (2). Poor metacognitive skills and poor social functioning have been demonstrated in patients with schizophrenia (14, 15). Impaired metacognitive skills are correlated with occupational impairment, low self-esteem and social anxiety (11, 16, 17). Metacognitive skills could therefore be key in translating cognitive performance into life skills. Schizophrenia presents the greatest metacognitive challenges among psychiatric illnesses (18–22). According to some studies, metacognitive difficulties do not significantly differ between patients with schizophrenia and people without psychiatric problems (23, 24). Other studies have observed greater deficits in metacognition among patients with schizophrenia compared to healthy subjects, particularly in forming complex ideas about themselves and others (20, 25, 26).

Alterations in social cognition have been widely demonstrated in schizophrenia (25, 26). These deficits are thought to be the most frequent and earliest impairments and may be the root of clinical symptom formation (27). Many studies have consistently documented significant impairments in several social cognitive domains in schizophrenia, such as theory of mind and emotion perception and processing, social perception and social knowledge (26, 28–30). These impairments may be as severe as or more severe than neuropsychological deficits (31, 32). Indeed, neurocognition was conceptualized as a necessary basis for social cognitive abilities (33). Thus, social cognition deficits may be present without neurocognitive impairments, whereas the opposite is rare (32). Among them, theory of mind is the most impaired function and the most strongly related to functional deficits (26, 34, 35).

The broadest definitions of metacognition appear to have considerable overlap with neurocognition or aspects of social cognition. Indeed, some authors argue that the ability to infer the emotional states and cognitions of others falls within the scope of metacognition (2), while other authors incorporate these processes into the definition of theory of mind (36). Although the extent of this overlap is still debated (36), several authors have argued that these are separate constructs (37, 38). Conceptually, metacognition includes the processing of internal information associated with social cues to enable synthesis. On the other hand, social cognition is a measure of performance because it assesses the accuracy of judgments of social cues (23). Beyond those conceptual differences, discrepancies in brain activation have been reported. Both self-referential thought processes and thinking about other people with similar thoughts lead to similar activation of the ventromedial prefrontal cortex. In contrast, thinking about the thoughts of people perceived as different from oneself activates a more dorsal region of the ventromedial prefrontal cortex, which is not involved in self-reflection processes (39).

Furthermore, clinical and cognitive symptoms in schizophrenia differ in their relationship to social cognition and metacognition. Negative symptoms have been correlated with metacognition and social cognition, whereas positive symptoms have been associated with only social cognition (14, 35). Neurocognition encompasses mental processes like thinking, problem-solving, memory, attention, and executive functions (40). Social cognition, especially theory of mind, may be more generally linked to neurocognition (41), whereas metacognition has been primarily associated with verbal memory (42, 43) and cognitive flexibility (44, 45).

In contrast, some studies have suggested that metacognitive skills are necessary to address social cognition abilities. During a social cognition task in an fMRI study, brain activations related to metacognitive skills preceded brain activations related to social cognition. These results suggest that metacognition is a lower-order process used for understanding others’ mental state (46). Reciprocally, social cognition abilities may serve as a basis for metacognitive activity. In particular, emotion processing may be required for metacognitive regulation and for integrating the representation of others (2). Mirror neurons are implicated in the conceptualization of others’ goals, meanings and intentions through simulation processes (47).

Since there are similarities and discrepancies between social cognition and metacognition on the conceptual, clinical and neurological levels in schizophrenia, elucidating the correlation between them seems essential. To the best of our knowledge, no systematic review has examined the correlation between social cognition and metacognitive outcomes in schizophrenia. To examine this correlation, we reviewed quantitative studies that compared metacognitive assessments to social cognition evaluations (theory of mind, emotion processing, attributional style, or social perception and knowledge) in adult outpatients with a schizophrenia spectrum disorder.

## Methods

In line with the PRISMA guidelines, a systematic search was performed of clinical trials published in English or French in the Medline, ScienceDirect, and PsycINFO databases from inception and extraction to April 30th, 2020 and updated to include articles published up to July 13th, 2023 following the same protocol. We searched the Medline, ScienceDirect, and PsycINFO databases with the same search strategy (see Appendix 1) using the following search terms to describe our population (“schizophrenia,” “psychosis,” “schizoaffective”), metacognitive outcome (“metacognition,” “metacognitive”) and social cognitive outcome (“social cognition,” “social cognitive,” “social intelligence,” “theory of mind,” “ToM,” “mentalizing,” “emotion processing,” “emotion perception,” “face perception,” “faces perception,” “social perception,” “attribution”). The aims, inclusion criteria, data collection and analysis of this review were specified in advance in the PROSPERO database (CRD42020160259).

For this review, we selected clinical trials assessing the correlation between social cognition and metacognitive outcomes in adult patients living with a schizophrenia spectrum disorder. First, we excluded reports that did not involve a study (e.g., reviews, letters to the editor), involved studies without results (e.g., presentations of a study design) and case studies. Moreover, we restricted our selection to peer-reviewed written communications such as articles and doctoral theses, excluding poster presentations, verbal communications, and chapters of books. No restrictions were applied regarding publication date. We considered all publications in English or French. We included studies with or without a control group, but we did not include control in the statistical analyses. Finally, studies needed to include at least one social cognitive measure and one metacognitive measure derived from a cognitive assessment, a rater-administered scale or a questionnaire. To assess whether a score was a social cognition or metacognitive measure, we searched the original article on the corresponding assessment or scale. All statistical analyses comparing those two scores were accepted.

Reports that were identified in the databases were compared on the basis of their author, title and Digital Object Identifier to eliminate duplicates. Reports were screened for eligibility on the basis of their title and abstract independently by the first two authors. Reports that clearly did not meet the inclusion criteria were excluded, and the remaining reports were then assessed independently by the same two authors on the basis of their full text. Multiple publications were combined when the same patient sample was used in two or more reports. Disagreements were resolved through discussion between the two authors. Finally, a manual search was conducted to identify potential additional reports.

Using a data extraction form, we collected relevant information from the selected studies, including sample characteristics, patient assessments, outcomes and study design. The first author extracted the data from the reports, and the second author confirmed the collected data. As needed, the authors of selected reports were contacted by email with the request to provide missing or complementary information.

Reports that compared a metacognitive measure with a social cognitive measure were included in the review. All reports using a correlation coefficient to examine the relationship between these measures were included in the meta-analysis as well. When a report did not specify the correlation value, we asked the authors to provide it; if the value was not provided, the report was excluded from the meta-analysis. Using the Meta-Essential Package for correlation coefficients in Microsoft Excel (48), we used a Fisher Z transformation of the correlation coefficients and weighted each report according to the number of statistical analyses performed. We computed a weighted summary two-tailed correlation coefficient, an I-squared statistic and a Q statistic for all included studies. Furthermore, we performed analyses for four different social cognitive domains (i.e., emotion processing, theory of mind, attribution, other social cognitive functions), retaining only reports with assessments related to these domains. For each analysis, a forest plot was generated. When I-squared and Q statistics suggested heterogeneity, subgroup and sensitivity analyses were performed to identify the potential source of discrepancy. Finally, publication bias was assessed with Egger regression and Begg and Mazumdar test, and a fail-safe number was computed.

Risks of bias were assessed with the Cochrane risk of bias tool, and a risk of bias form with nine variables was completed by the first two authors. Disagreements between the two authors were resolved through discussion. Risks of bias were assessed using a classification table (see Supplementary Table S1). The risk of bias for medication was considered low when the participants’ treatment was stable for at least 15 days and homogeneous in the population (i.e., same class of medication). The risk was considered medium when the medication was either stable or homogeneous and was considered high if none of these criteria was met or if no information on medication was provided by the authors. The risk of bias in the blinding of the metacognition rating was considered low if the investigator performing the metacognitive assessment was blind to the participants’ social cognitive performance. This risk was considered high if metacognition and social cognition assessments were administered or rated by the same person or by two investigators without blinding. Concerning the validation of the assessments, the risk of bias was rated as follows: (a) low when metacognition and social cognition assessments were validated in the language of the participant in a previous study; (b) medium if the assessments were only translated, without a validation study; and (c) high if at least one study was neither validated nor translated. The risk of bias for ethical committee approval was considered low if the study was approved by any local or national ethical committee. Finally, the risk of bias for the outcome was considered: (a) low when the correlation between social cognition and metacognition was the primary outcome of the study; (b) medium when this correlation was a secondary outcome; and (c) high when the correlation was the subject of an ancillary study and not a part of the outcomes of the main study.

## Results

### Study selection

**Figure 1:**
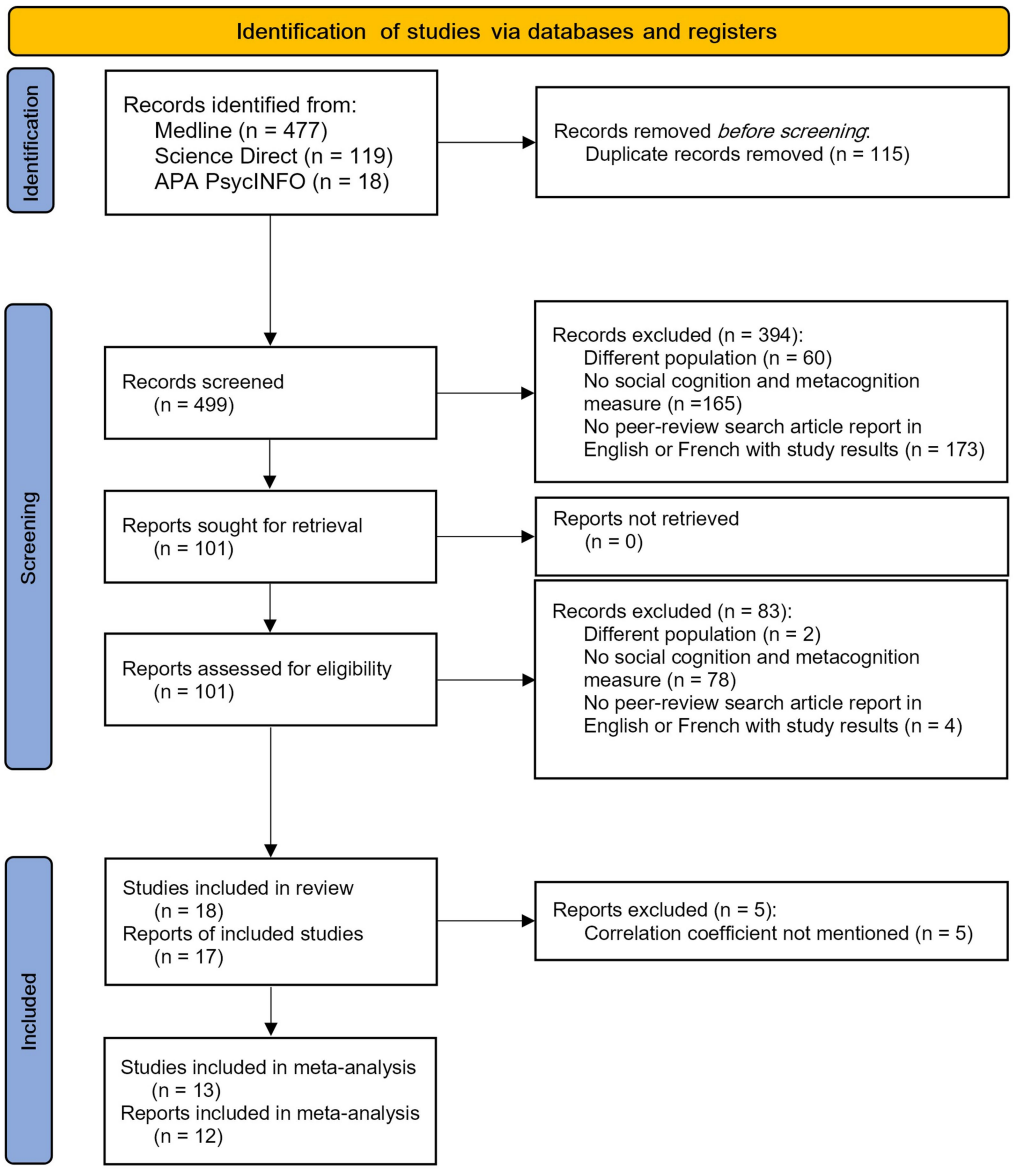
Report selection process.

The selection process is described in Figure 1. The search of the Medline, ScienceDirect and PsycINFO databases provided a total of 609 studies. No additional reports were found through a manual search. After removing duplicates, 494 publications remained. After reviewing the title and the abstract, 394 reports were discarded because it appeared that these studies did not meet the inclusion criteria (different population, no social cognition or metacognitive measure, not peer reviewed, missing study results).

In total, 83 reports were excluded because the full text did not meet the inclusion criteria. Among them, one report was initially included in the review because it used the Interpersonal Reactivity Index, which appeared to assess social cognition, particularly theory of mind (49). We finally excluded this report because the “perspective taking” measure assesses the tendency to take another person’s perspective (i.e., cognitive empathy) rather than the cognitive ability to do so. Another report was excluded from the review because the authors used an experimental social cognition task with a metacognitive measure; as such, the metacognitive and social cognitive measures were not independent (50). Finally, a report was excluded from the review because the metacognitive and social cognitive measures were extracted from the same experimental task (51).

One report included two distinct populations with schizophrenia spectrum disorders (52), and thus was considered two studies. Consequently, our analysis comprises a total of 18 studies and 17 reports included in the systematic review (see Appendix 2). Five reports were excluded from the meta-analysis because they did not report a correlation coefficient or did not provide results for all metacognition subscales. We contacted five authors because we were missing correlation coefficients. One author provided further information, and we were able to include their study in the meta-analysis (53). The other authors did not answer, and we only included their reports in the review (17, 54–56).

### Study characteristics

The characteristics of the included studies are presented in Supplementary Table S2. The reports selected for the meta-analysis were published in English between 2008 and 2021. The 18 studies included a total of 1,036 patients. Included participants had first-episode psychosis (FEP) in two studies, schizophrenia in four studies and schizophrenia spectrum disorder in 12 studies. Seven reports had a control group, with a total of 147 healthy volunteers, 30 patients with major depression, 30 patients with autism spectrum disorder and 58 patients with substance use disorder. We included two prospective studies, and the remaining eight studies were cross-sectional.

The included studies used 14 social cognition assessments and two metacognition questionnaires (see Table 1). Twelve studies used emotion processing assessments: the Bell Lysaker Emotion Recognition Task (BLERT), the Derntl task, the Face Emotion Identification Task (FEIT), the Eckman 60 Faces Test or the Tool for Recognition of Emotions in Neuropsychiatric DisorderS (TRENDS). The BLERT uses videos depicting professional interpersonal situations where the participant must recognize the emotion of the main character (57). The Derntl (58), FEIT (59), Eckman 60 Faces Test (60) and TRENDS (61) are four performance tasks in which the participant identifies facial emotions.

**Table 1:** Social cognitive and metacognitive assessments.

Cognitive functions	Assessment	Author
Theory of mind	Hinting Test	Corcoran et al., 1995
	Picture Sequencing Task	Brüne, 2003
	Reading the Mind in the Eyes Test (RMET)	Baron-Cohen, 2001
	Yoni Task	Shamay-Tsoory et al., 2007
	Movie for the Assessment of Social Cognition (MASC)	Dubreucq et al., 2022
Emotion processing	Face Emotion Identification Task (FEIT)	Kerr et al., 1993
	Derntl Task	Derntl, 2009
	Bell Lysaker Emotion Recognition Task (BLERT)	Bell et al., 1997
	Ekman 60 Faces Test	Young et al., 2002
	Tool for Recognition of Emotions in Neuropsychiatric DisorderS (TRENDS)	Behere et al., 2008
Attribution	Social Attribution Task - Multiple Choice (SAT-MC)	Klin, 2000
	Social Cognition and Object Relations Scale (SCORS)	Westen et al., 1990
Social cognition	Matrics Consensus Cognitive Battery - Social Cognition (MCCB-SC)	Nuechterlein et al., 2008
	Faux-Pas Task	Stone et al., 1998
Metacognition	Metacognitive Assessment Scale - Abbreviated (MAS-A)	Semerari et al., 2003
	Beck Cognitive Insight Scale (BCIS)	Beck et al., 2004

Nine studies used theory of mind assessments: the Hinting Test, the Reading the Mind in the Eyes Test (RMET), the Picture Sequencing Task, the Movie for the Assessment of Social Cognition (MASC) and the Yoni task. In the Hinting test, the participant indicates the implied intention of characters in stories (62). In the RMET, the participant infers the state of mind or thoughts of a person while seeing only their eyes (63). The Picture Sequencing Task uses cartoon strips where characters collaborate or betray one another; the participant sorts the strips to create a story (64). The Yoni task assesses affective and cognitive, first-order and second-order theory of mind using a cartoon character called Yoni; the participant guesses what Yoni thinks or likes using Yoni’s facial expressions (65). The MASC assesses theory of mind using a 15-min film depicting four individuals, with participants answering questions about the mental state of these individuals (66).

Three studies used attribution assessments: the Social Attribution Task – Multiple Choice (SAT-MC) and the Social Cognition and Object Relations Scale – Understanding Social Causality subscale (SCORS-USC). In the SAT-MC, the participant guesses the purpose of a geometric shape mimicking social interactions with other shapes (67). The SCORS-USC assesses the accuracy of the attribution of intention in a participant’s Thematic Apperception Test narratives (68).

Four studies used other social cognitive assessments: the Social Cognition subscale of the MATRICS Consensus Cognitive Battery (MCCB-SC) or the Faux-Pas Task. The MCCB-SC is an emotional intelligence test that assesses the ability to manage emotions (69). The Faux-Pas Task assesses the ability to identify social missteps and the consequences for others’ mental states throughout ten stories (70).

The 18 studies provided 84 correlations in total (see Supplementary Table S2). Fifteen studies used the Metacognitive Assessment Scale – Abbreviated (MAS-A), including ten that correlated the total score with social cognitive measures. The MAS-A consists of four scales that assess four metacognitive processes: Self-Reflectivity (MAS-SR), the ability to understand Other’s mind (MAS-O), Decentration (MAS-D) and Mastery (MAS-M). Self-reflectivity refers to the ability to generate representations of one’s self, decentration is the ability to understand the environment from different perspectives, and mastery is the ability to implement effective strategies to accomplish cognitive tasks and regulate one’s behavior (2).

(a) In the eight studies that examined the correlation of the MAS-A total score with emotion processing assessments, the MAS-A was significantly correlated with emotion processing. The correlation was positive in seven studies and negative in one study (52). One study examined the correlation of MAS-A subscale scores with the FEIT and observed similar results (71). However, in the three other studies that used MAS-A subscales, the results were heterogeneous: one study observed significant correlations between the BLERT and MAS-A subscale scores except for the MAS-D (53); one study found significant correlations between the Derntl task and MAS-SR scores but heterogeneous results for the other subscales depending on the emotion assessed (72); and one study observed no significant correlation between the TRENDS and MAS-SR or MAS-O scores (54). Finally, one report used MAS-A total scores and repeated exploratory partial correlation analyses with MAS-A subscale scores; the authors observed significant correlations between the Ekman 60 Faces Test scores and MAS-SR, MAS-O and MAS-D scores (52).

(b) The results of the studies were heterogeneous for theory of mind and attribution. Three studies assessed the correlation between scores on the MAS-A and the Hinting Task, one of which reported a significant positive correlation (19). Interestingly, the study that found a significant correlation included patients with a higher mean level of education (16.64 years) than the two other studies (12.66 and 12.88 years). In addition, one study used MAS-A subscale scores and observed a significant correlation only for the MAS-SR score (53). Three studies used other assessments and found positive correlations between the MAS-A score and theory of mind. One study reported a significant correlation between scores on the MAS-A and the RMET (19). The two other studies described significant correlations of scores on the MAS-SR and MAS-O with the RMET (73) or the Picture Sequencing Task (53). One study reported a significant correlation between the MAS-A score and attribution (74), and one study found nonsignificant results (75). Furthermore, the study that used MAS-A subscale scores reported a significant correlation only for MAS-D scores.

(c) Two studies used the MCCB-SC score and described significant correlations with MAS-A scores (75) and with the MAS-SR and MAS-M scores but not the MAS-O and MAS-D scores (53). Finally, the only study that used the Faux-Pas Task found significant correlations of this score with only the MAS-SR and MAS-O scores (71).

Four studies reported nonsignificant correlations between the Beck Cognitive Insight Scale (BCIS) score and social cognitive measures. The BCIS is a self-rated questionnaire with two subscales: (a) Self-Reflectiveness (BCIS-SR), which assesses the ability to observe one’s own mental production and consider different explanations; and (b) Self-Certainty (BCIS-SC), which assesses overconfidence in the validity of one’s own beliefs (76). Two studies reported both subscales, one used only the BCIS-SR, and one study generated a composite score using the two subscales (24). One additional study used the BCIS but did not report any correlations (55).

Finally, six studies provided complementary results. The description of regression analyses, additional correlations and group comparisons in the included studies can be found in Supplementary material S1.

### Meta-analysis

Random effect analysis of the 13 studies showed a significant weighted summary correlation coefficient (*r* = 0.28, 95% CI: [0.14, 0.41], *z* = 4.18, *p* < 0.001), as shown in Table 2. *I^2^* and *Q* values (*I^2^* = 60.80%; *Q* (11) = 33.16, *p* = 0.002) suggested heterogeneity. A statistically significant effect was observed when comparing the 11 studies that used the MAS-A and the two studies that used the BCIS in a fixed-effect subgroup analysis (*Q* (12) = 33.30, *p* = 0.001), with a significant correlation coefficient for MAS-A studies (*r* = 0.31, 95% CI: [0.23, 0.38], *p* = 0.001) and a non-significant correlation coefficient for BCIS studies (*r* = 0.15, 95% CI: [−0.85, 0.92], *p* = 0.513). Due to the discrepancy in social cognition assessments, we performed four secondary analyses to assess the correlations among theory of mind, emotion processing, attribution and other social cognitive components.

**Table 2:** Funnel plot representing the weighted correlation of each study included in the meta-analysis, with respective weight (*n* = 12).

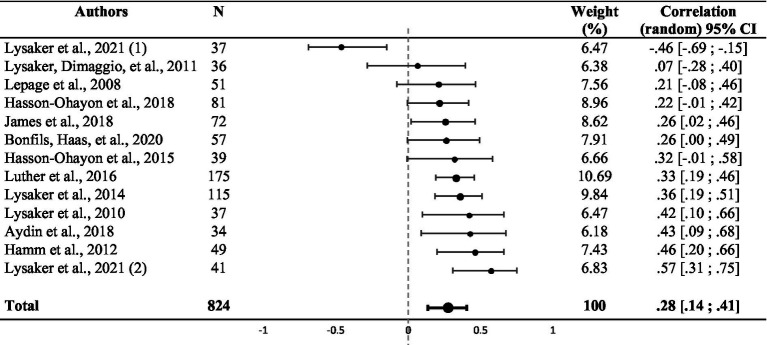

(1) Early schizophrenia spectrum disorders population; (2) Prolonged schizophrenia population.

There was a significant weighted summary correlation coefficient (*r* = 0.23, 95% CI: [0.11, 0.34], *z* = 5.22, *p* < 0.001) for the five studies that used theory of mind assessments. *I^2^* and *Q* values (*I^2^* = 0.00%; *Q* (4) = 2.61, *p* = 0.625) suggested no heterogeneity (see Table 3). Random effect analysis of the three studies that used attribution assessments revealed a significant weighted summary correlation coefficient (*r* = 0.25, 95% CI: [−0.03, 0.49], *z* = 3.90, *p* < 0.001). *I^2^* and *Q* values (*I^2^* = 0.00%; *Q* (2) = 1.56, *p* = 0.458) suggested no heterogeneity (see Table 4). Ten studies assessed the correlation between emotion processing and metacognition. Random effect analysis revealed a significant weighted summary correlation coefficient (*r* = 0.29, 95% CI: [0.07, 0.48], *z* = 3.02, *p* = 0.002), as shown in Table 5. *I^2^* and *Q* values (*I^2^* = 77.16%; *Q* (9) = 39.40, *p* < 0.001) suggested heterogeneity. No statistically significant effect was observed when comparing the six studies that used the BLERT and the four studies that used another emotion processing assessment in a fixed-effect subgroup analysis (*Q* (9) = 9.25, *p* = 0.42, see Supplementary Table S3). A sensitivity analysis excluding the study with atypical populations (52) revealed a significant weighted summary correlation coefficient (*r* = 0.34, 95% CI: [0.22, 0.45], *z* = 6.42, *p* < 0.001) with lower heterogeneity (*I^2^* = 39.88%, *Q* (8) = 11.64, *p* = 0.113). Random effect analysis of the four studies that used other social cognition assessments showed a significant weighted summary correlation coefficient (*r* = 0.26, 95% CI: [0.18, 0.33], *z* = 10.33, *p* < 0.001). *I^2^* and *Q* values (*I^2^* = 0.00%; *Q* (3) = 0.45, *p* = 0.93) suggested no heterogeneity (see Table 6).

**Table 3:** Funnel plot representing the weighted correlation of each study included in the meta-analysis and using theory of mind assessment, with respective weight (*n* = 5).

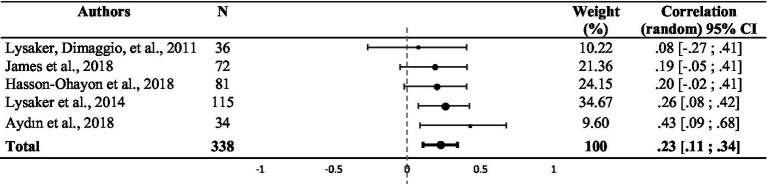

**Table 4:** Funnel plot representing the weighted correlation of each study included in the meta-analysis and using attribution assessment, with respective weight (n = 3).

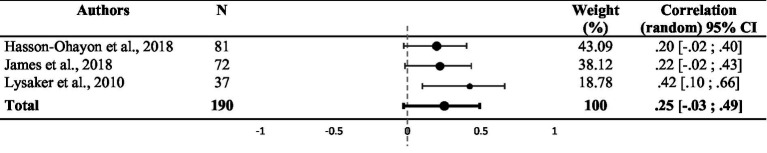

**Table 5:** Funnel plot representing the weighted correlation of each study included in the meta-analysis and using emotion processing assessment, with respective weight (*n* = 9).

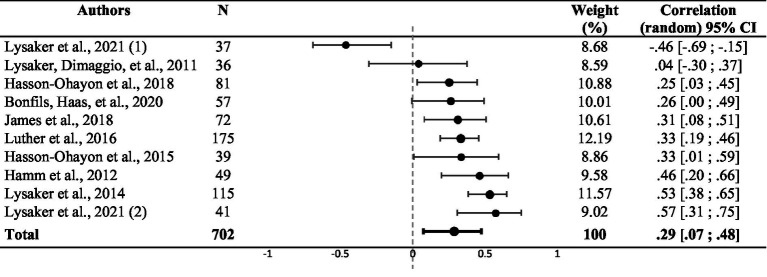

(1) Early schizophrenia spectrum disorders population; (2) Prolonged schizophrenia population.

**Table 6:** Funnel plot representing the weighted correlation of each study included in the meta-analysis and using other social cognitive assessment, with respective weight (*n* = 4).

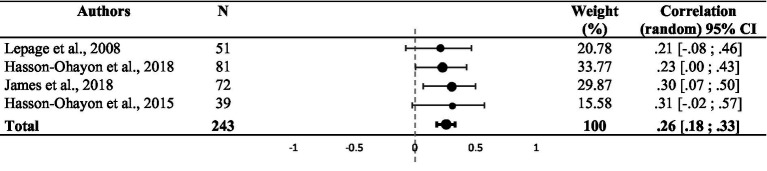

**Figure 2:**
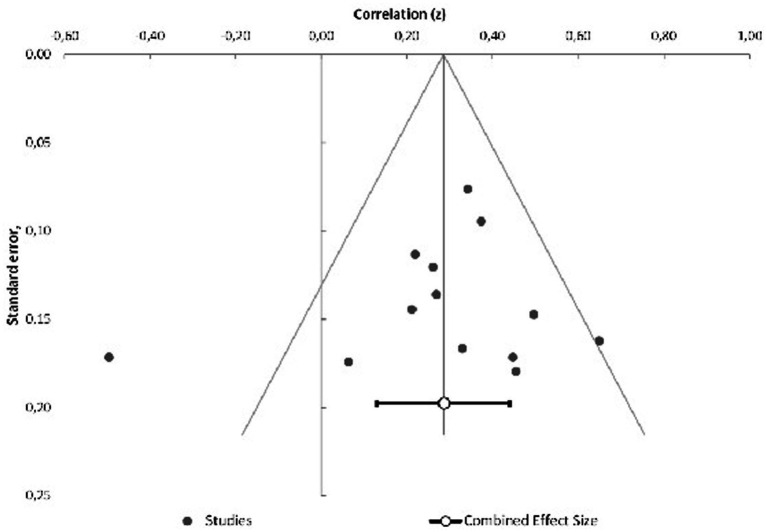
Funnel plot of the meta-analysis of published studies.

The description of risk of bias analysis in the included studies can be found in Supplementary material S2 and presented in Supplementary Table S4. The funnel plot for the 13 studies included in the meta-analysis is shown in Figure 2. Egger regression (*t* = −0.50, *p* = 0.63) and Begg and Mazumdar test for rank correlation (*z* = 0.06, *p* = 0.95) indicated no evidence of publication bias. Additionally, the fail-safe number was 85, which is considered large (77). The results of Egger regression and Begg and Mazumdar test for each social cognitive function are shown in Supplementary Table S5. Possible publication bias was identified for the correlation between attribution and metacognition (Egger regression: *t* = 26.78, *p* = 0.02).

## Discussion

To our knowledge, the present meta-analysis is the first to examine the correlation between metacognition and social cognition in schizophrenia. This systematic review included 1,036 participants in 17 reports published between 2008 and 2023. Twelve reports were included in the meta-analysis, with a total of 824 participants. There was a significant positive weighted summary correlation between social cognition and metacognition assessments in individuals with a schizophrenia spectrum disorder. The correlation coefficient was 0.28, which indicates a small to medium effect size (78). There was no evidence of publication bias, but we found some heterogeneity among studies.

### Are metacognition and social cognition correlated in schizophrenia?

Seven reports described only significant correlations between social cognition and metacognition, six of which were included in the meta-analysis. No individual risk of bias seemed to differentiate those reports from the ones with nonsignificant results. However, the reports with significant results were among those with overall lower risks of bias. Two of them were the only reports with a low risk of bias for medication. Moreover, all of them had independent and blinded metacognition and social cognition assessments. Furthermore, one of those reports was an ancillary study. These data strengthen the robustness of the main result. On the other hand, one report included in the meta-analysis and three additional reports included in the review found only nonsignificant correlations. Of note, two of those reports had a high risk of bias for medication, three of them had a high risk of bias for blindness of assessments and two had a high risk of bias for the validation of the assessment.

The small-to-medium correlation between social cognition and metacognition in participants with a schizophrenia spectrum disorder is consistent with theories that postulate that these factors represent two semi-independent domains of cognition (9). As previously stated, social cognition and metacognition are related to distinct activations of the same brain regions in healthy volunteers (39). Tamir and Mitchel (46) proposed a hierarchical relationship between social cognition and metacognition, suggesting that inferring the judgment on another person requires reflection on one’s own judgment followed by a series of adjustments. On the contrary, Kukla and Lysaker (79) have posited that social cognition is a lower-order process needed for metacognition. Another explanation for this range of correlation coefficients is that those two domains are interrelated with neurocognition. A longitudinal study found that improvements in metacognition were associated with improvements in neurocognition and social cognition, indicating parallel trajectories for these three domains (79). In schizophrenia, the correlation between social cognition and metacognition could be mediated by neurocognition and symptoms (80).

The discrepancy in the main analysis could be explained by the metacognition assessments that were used. Indeed, included studies only used two metacognitive assessments, the BCIS and the MAS-A. The correlation coefficients with social cognition were statistically different for these two assessments, with a positive correlation for MAS-A and no correlation for BCIS. It is possible that cognitive insight (assessed by the BCIS) is distinct from metacognition (assessed by the MAS-A). Four studies in the review examined the correlation of BCIS scores with social cognition. Regardless of use of the BCIS-SR score, BCIS-SC score or a composite score, no correlation was identified with social cognition. The BCIS was designed within the framework of cognitive theory and derived “cognitive insight” from the concept of “insight,” defined as the awareness of one’s own mental illness (76). As such, the BCIS defines cognitive insight as the ability of people living with schizophrenia to appraise and correct misinterpretations or distorted beliefs that can occur. In contrast, the MAS-A total score assesses metacognition which is understood as the ability to monitor one’s mental state and regulate behavior, regardless of the occurrence of delusional beliefs and thoughts (2). The MAS-A also considers metacognition the capacity to form and integrate complex representations of the self and others. These variables could represent two ends of a spectrum, ranging from discrete metacognitive activities, assessed primarily by the BCIS, to synthetic metacognition, assessed by the MAS-A (81). Our results suggest that social cognition would then be correlated with general mentalizing abilities in schizophrenia and not with a more specific reflection upon distorted or delusional experiences. The fact that the BCIS is a self-rated questionnaire, in contrast to the MAS-A, can also explain the lack of correlation between the BCIS score and social cognitive measures. Indeed, it is possible that patients with the same level of cognitive insight scored differently on the BCIS because of a different subjective judgment or understanding of the sentences of the scale.

The results also emphasize that social cognition may exhibit different correlations according to the metacognitive dimension assessed. Indeed, the MAS-SR score was more frequently correlated with social cognitive measures than other MAS subscale scores. This result is in line with our main result because the ability to recognize and define one’s own emotions and cognitive processes seems to be the closest to metacognition as defined by Flavell (1). In contrast, the MAS-M score exhibited the weakest correlations with social cognitive measures in the review. Mastery (i.e., implementation of cognitive strategies and behavioral regulation) was the most impaired metacognitive function in previous work (11). Our results may be due to a floor effect for mastery scores in the included studies, which reduced the range of the data. Conversely, this lack of significant correlations may originate from an indirect relationship between social cognition and mastery that is mediated by quality of life. Indeed, improvements in social cognition and quality of life over time can significantly predict improvements in self-reflectivity and mastery (79). Surprisingly, the relationship between the ability to understand the mental states of others and social cognition has been inconsistent in the literature. Some authors suggest that this inconsistency is tied to the development of the MAS-A (72). The MAS-SR and MAS-O subscales are rated by assessing first the cognitive component and then the emotional component of each subdimension. Furthermore, the MAS-O assesses the tendency to address others’ thoughts or emotions in reasoning activities rather than the ability to do so effectively. In contrast, social cognitive assessments provide performance-based measures. People living with schizophrenia could continue to address the thoughts and emotions of others despite impairments in the ability to do so.

Overall, these results are consistent with the hypothesis of metacognition as a modular skill composed of related but functionally independent subfunctions (2). This is also consistent with findings that metacognitive subdimensions are associated with different neurocognitive functions or exhibit different correlations with quality of life in schizophrenia (43, 71).

### Is metacognition correlated with emotion processing?

The most studied social cognitive component in the meta-analysis was emotion processing. The results suggest a significant small-to-medium correlation between metacognition and emotion processing in schizophrenia. Metacognition and emotion processing are conceptually associated because emotion processing is thought to be a key component in metacognitive mastery and understanding of others’ minds (2). Furthermore, a previous study on the metacognition of emotion recognition in neurodegenerative diseases suggested that emotion processing and metacognitive impairments share cerebral substrates (i.e., amygdala, insula, frontal and temporal regions) (82).

There was a discrepancy among studies that was not attributable to the emotion recognition assessment used but rather partly due to inclusion of an atypical population in one study. Lysaker et al. included one group of 37 patients with early psychosis (i.e., 5 years of illness and three episodes or less) and one group of 41 patients meeting the criteria for schizophrenia for a minimum of 6 years (52). The correlation between the MAS-A score and the emotion processing assessment was significant in both groups; however, this correlation was positive for the schizophrenia group and negative for the early psychosis group. Surprisingly, this was the only study in the meta-analysis that reported a significant negative correlation between social cognition and metacognition, even though two other studies in the review included patients with first-episode psychosis. Although the emotion processing assessment does not seem to explain the discrepancy in the results, it is noteworthy that these assessments greatly differed from one another. Some of these assessments used pictures of faces displaying emotions, while others used video sequences, which provide more visual and verbal cues. The participants may have to name the emotion among two or six propositions, or they may have to guess the emotional face of someone in a social situation. Some tests use the six basic emotions (i.e., happiness, fear, surprise, anger, disgust, sadness), while others use shame instead of disgust. The emotions used in the assessments could be crucial because different emotions may not need the same level of metacognition. Indeed, in the study by Bonfils et al. (72), disgust was correlated with all MAS dimensions, while anger was only correlated with self-reflectivity.

### Is metacognition correlated with theory of mind and attribution?

There was a significant positive weighted summary correlation of metacognition with theory of mind and attribution. The effect sizes of the correlation coefficients were small to medium with no evidence of heterogeneity in the meta-analysis. Theory of mind and attribution share conceptual similarities and have been associated in previous work (83); consistent with these findings, they share similar correlations with metacognition. Nevertheless, the review identified differences among studies. Three studies assessed the correlation between scores on the MAS-A and the Hinting Task, and only one of them reported a significant positive correlation (19). In contrast, the two studies that assessed the correlation between scores on the MAS-A and the RMET, as well as the study that used the Picture Sequencing Task, found significant results. The Hinting Task assesses verbal theory of mind, while the RMET and the Picture Sequencing Task use nonverbal material. This result is perplexing, no difference has been identified between verbal and nonverbal theory of mind performance in schizophrenia in previous studies (29). The distinction between verbal and nonverbal theory of mind requires further investigation for definitive conclusions.

### Limits and future direction

Our meta-analysis has three main limitations. First, we observed selective reporting of quantitative data in several studies included in the review. This suggests that the correlations included in the meta-analysis do not encompass the entirety of scientific data on the subject. However, our results indicate no risk of publication bias across the whole meta-analysis and only a possible risk of publication bias for attribution studies. Furthermore, we performed subgroup analyses to better understand the correlation between metacognition and each social cognitive function. Subgroup analyses in meta-analyses lack statistical power, and their results should be interpreted with caution (84), especially in meta-analyses that include only a few studies. Finally, we found that studies used a wide variety of tests to assess social cognition, either as a whole or in specific components. We observed different results when the test assessed “hot” (i.e., emotional) or “cold” (i.e., cognitive) social cognition. For instance, in the same population, James et al. (75) found that emotional social cognition assessments (emotion recognition and emotion management) were correlated with metacognition, while more cognitive assessments of social cognition (cognitive theory of mind and attribution) were not correlated with metacognition. Other studies support the distinction between cognitive and affective processes when thinking about others (85). Emotional and cognitive social cognition may be understood as separate processes (86). Future studies on the association between metacognition and social cognition may differentiate between social cognition processes rather than social cognitive functions. It would be particularly interesting to compare the correlation between metacognition and cognitive or affective theory of mind. Comparing the correlation between metacognition and verbal or nonverbal social cognition assessments may also contribute to a better understanding of the relationship between those constructs. Furthermore, our results emphasize the importance of addressing metacognitive impairments, as it is well-established that interventions targeting metacognition have a positive impact on social cognitive difficulties. However, the extent of their transfer to daily life remains to be demonstrated (87).

Further research is needed on the correlation between attribution and metacognition, as there were a limited number of studies on the subject. Future studies may also use other metacognition assessments to better understand the different patterns of correlations between scores on the MAS-A and the BCIS observed in this review. In this regard, the metacognition questionnaire may be an interesting assessment because it is a self-rated questionnaire (such as the BCIS) but considers metacognition as a thinking style regarding one’s own thought processes, which is closer to the MAS-A definition of metacognition (81). Other scales used in the field of educational psychology to assess metacognitive monitoring and regulation during problem solving (88), such as the metacognitive assessment inventory, could also be adapted in psychiatric populations and compared to social cognition measures. Alternatively, systematic reviews in the future could refine their inclusion criteria to target schizophrenia or FEP. Emphasis could also be placed on a social cognition component such as theory of mind.

## Conclusion

Our systematic review and meta-analysis that adhered to PRISMA guidelines indicated a significant correlation between social cognition and metacognition in individuals with a schizophrenia spectrum disorder. This result was replicated with three social cognitive domains: theory of mind, attribution and emotion processing. In contrast, we observed different patterns of correlation for different metacognitive concepts or components (i.e., cognitive insight, self-reflectivity, understanding others’ minds, decentration, mastery). These results are in line with the theory that social cognition and metacognition are two distinct but interrelated constructs.

The association between metacognition and social cognition, as well as neurocognition, needs to further study to better identify and treat the cognitive symptomatology of schizophrenia. This meta-analysis included studies reporting correlations rather than causal relationships between metacognition and social cognition. Nonetheless, our results are in line with previous work that stressed the need to treat metacognitive impairments to improve other spheres of cognition and psychosocial functioning in patients with schizophrenia (81). Metacognitive training (89) or metacognitive reflection and insight therapy (90) are two nonpharmaceutical interventions that target discrete or synthetic metacognition. Oxytocin may also be a promising treatment to improve metacognition and social cognition (91). Improvements in social cognition over time are positively correlated with improvements in metacognition (79). Thus, enhancing social cognition with cognitive remediation programs could potentially be beneficial for the broad network of cognition. Finally, individuals living with a schizophrenia spectrum disorder may benefit from an integrated cognitive remediation approach that addresses neurocognition, social cognition and metacognition in its discrete and synthetic aspects.

## Data availability statement

The original contributions presented in the study are included in the article/Supplementary material, further inquiries can be directed to the corresponding author.

## Author contributions

AM: Conceptualization, Investigation, Methodology, Project administration, Supervision, Validation, Writing – original draft, Writing – review & editing. CI: Conceptualization, Data curation, Formal analysis, Investigation, Methodology, Supervision, Validation, Writing – review & editing, Software. M-CC: Writing – review & editing. DJ: Writing – review & editing.
