# Supplementary material for: Link between metacognition and social cognition in schizophrenia: a systematic review and meta-analysis
Source: Front Psychiatry. 2023 Dec 22;14:1285993. doi: 10.3389/fpsyt.2023.1285993 (PMC10766774; doi:10.3389/fpsyt.2023.1285993)
Supplement: Supplementary file 1 [file Data_Sheet_1.docx]

**Link between metacognition and social cognition in schizophrenia: a systematic review and meta-analysis** – **SUPPLEMENTARY MATERIALS**

***APPENDIX* 1**

Search strategy for MEDLINE, with results on July 13^th^, 2023.

1. "schizophrenia" AND "metacognition" AND "social cognition" (result: 163)
2. "schizophrenia" AND "metacognition" AND "theory of mind" (result: 74)
3. "schizophrenia" AND "metacognition" AND "ToM" (result: 19)
4. "schizophrenia" AND "metacognition" AND "emotion processing" (result: 32)
5. "schizophrenia" AND "metacognition" AND "social perception" (result: 45)
6. "schizophrenia" AND "metacognition" AND "attribution" (result: 34)
7. "schizophrenia" AND "metacognition" AND "social intelligence" (result: 29)
8. "schizophrenia" AND "metacognition" AND "face perception" (result: 13)
9. "schizophrenia" AND "metacognition" AND "faces perception" (result: 5)
10. "schizophrenia" AND "metacognition" AND "mentalizing" (result: 213)
11. "schizophrenia" AND "metacognition" AND "social cognitive" (result: 185)
12. "schizophrenia" AND "metacognition" AND " emotion perception” (result: 25)
13. "schizophrenia" AND "metacognitive" AND "social cognition" (result: 150)
14. "schizophrenia" AND "metacognitive" AND "theory of mind" (result: 72)
15. "schizophrenia" AND "metacognitive" AND "ToM" (result: 19)
16. "schizophrenia" AND "metacognitive" AND "emotion processing" (result: 32)
17. "schizophrenia" AND "metacognitive" AND "social perception" (result: 44)
18. "schizophrenia" AND "metacognitive" AND "attribution" (result: 33)
19. "schizophrenia" AND "metacognitive" AND "social intelligence" (result: 29)
20. "schizophrenia" AND "metacognitive" AND "face perception" (result: 13)
21. "schizophrenia" AND "metacognitive" AND "faces perception" (result: 4)
22. "schizophrenia" AND "metacognitive" AND "mentalizing" (result: 217)
23. "schizophrenia" AND "metacognitive" AND "social cognitive" (result: 184)
24. "schizophrenia" AND "metacognitive" AND " emotion perception” (result: 24)
25. "psychosis" AND "metacognition" AND "social cognition" (result: 80)
26. "psychosis" AND "metacognition" AND "theory of mind" (result: 41)
27. "psychosis" AND "metacognition" AND "ToM" (result: 12)
28. "psychosis" AND "metacognition" AND "emotion processing" (result: 23)
29. "psychosis" AND "metacognition" AND "social perception" (result: 24)
30. "psychosis" AND "metacognition" AND "attribution" (result: 18)
31. "psychosis" AND "metacognition" AND "social intelligence" (result: 15)
32. "psychosis" AND "metacognition" AND "face perception" (result: 0)
33. "psychosis" AND "metacognition" AND "faces perception" (result: 1)
34. "psychosis" AND "metacognition" AND "mentalizing" (result: 162)
35. "psychosis" AND "metacognition" AND "social cognitive" (result: 103)
36. "psychosis" AND "metacognition" AND "emotion perception" (result: 15)
37. "psychosis" AND "metacognitive" AND "social cognition" (result: 75)
38. "psychosis" AND "metacognitive" AND "theory of mind" (result: 37)
39. "psychosis" AND "metacognitive" AND "ToM" (result: 9)
40. "psychosis" AND "metacognitive" AND "emotion processing" (result: 23)
41. "psychosis" AND "metacognitive" AND "social perception" (result: 23)
42. "psychosis" AND "metacognitive" AND "attribution” (result: 18)
43. "psychosis" AND "metacognitive" AND "social intelligence" (result: 15)
44. "psychosis" AND "metacognitive" AND "face perception" (result: 5)
45. "psychosis" AND "metacognitive" AND "faces perception" (result: 0)
46. "psychosis" AND "metacognitive" AND "mentalizing" (result: 161)
47. "psychosis" AND "metacognitive" AND "social cognitive" (result: 104)
48. "psychosis" AND "metacognitive" AND "emotion perception" (result: 14)
49. "schizoaffective" AND "metacognition" AND "social cognition" (result: 6)
50. "schizoaffective" AND "metacognition" AND "theory of mind" (result: 3)
51. "schizoaffective" AND "metacognition" AND "ToM" (result: 0)
52. "schizoaffective" AND "metacognition" AND "emotion processing" (result: 0)
53. "schizoaffective" AND "metacognition" AND "social perception" (result: 1)
54. "schizoaffective" AND "metacognition" AND "attribution" (result: 2)
55. "schizoaffective" AND "metacognition" AND "social intelligence" (result: 1)
56. "schizoaffective" AND "metacognition" AND "face perception" (result: 1)
57. "schizoaffective" AND "metacognition" AND "faces perception" (result: 0)
58. "schizoaffective" AND "metacognition" AND "mentalizing" (result: 15)
59. "schizoaffective" AND "metacognition" AND "social cognitive" (result: 4)
60. "schizoaffective" AND "metacognition" AND "emotion perception" (result: 0)
61. "schizoaffective" AND "metacognitive" AND "social cognition" (result: 6)
62. "schizoaffective" AND "metacognitive" AND "theory of mind"(result: 2)
63. "schizoaffective" AND "metacognitive" AND "ToM" (result: 0)
64. "schizoaffective" AND "metacognitive" AND "emotion processing" (result: 0)
65. "schizoaffective" AND "metacognitive" AND "social perception" (result: 1)
66. "schizoaffective" AND "metacognitive" AND "attribution" (result: 2)
67. "schizoaffective" AND "metacognitive" AND "social intelligence" (result: 1)
68. "schizoaffective" AND "metacognitive" AND "face perception" (result: 1)
69. "schizoaffective" AND "metacognitive" AND "faces perception" (result: 0)
70. "schizoaffective" AND "metacognitive" AND "mentalizing" (result: 14)
71. "schizoaffective" AND "metacognitive" AND "social cognitive" (result: 7)
72. "schizoaffective" AND "metacognitive" AND "emotion perception" (result: 0)

***APPENDIX* 2**

References of all articles included in the meta-analysis and review

1. Aydın, O., Lysaker, P. H., Balıkçı, K., Ünal-Aydın, P., & Esen-Danacı, A. (2018). Associations of oxytocin and vasopressin plasma levels with neurocognitive, social cognitive and meta cognitive function in schizophrenia. *Psychiatry Research*, *270*, 1010–1016.
2. Bonfils, K. A., Haas, G. L., & Salyers, M. P. (2020). Emotion-specific performance across empathy tasks in schizophrenia: Influence of metacognitive capacity. *Schizophrenia Research: Cognition*, *19*, 100139.
3. Dubreucq, J., Martin, A., Gabayet, F., Plasse, J., Wiesepape, C., Quilès, C., ... & Lysaker, P. H. (2022). Contrasting the social cognitive and metacognitive capacities among patients with schizophrenia and autism spectrum disorders enrolled in psychiatric rehabilitation. *The Journal of Nervous and Mental Disease*, 10-1097.
4. Hamm, J. A., Renard, S. B., Fogley, R. L., Leonhardt, B. L., Dimaggio, G., Buck, K. D., & Lysaker, P. H. (2012). Metacognition and social cognition in schizophrenia: Stability and relationship to concurrent and prospective symptom assessments. *Journal of Clinical Psychology*, *68*(12), 1303–1312.
5. Hasson-Ohayon, I., Avidan-Msika, M., Mashiach-Eizenberg, M., Kravetz, S., Rozencwaig, S., Shalev, H., & Lysaker, P. H. (2015). Metacognitive and social cognition approaches to understanding the impact of schizophrenia on social quality of life. *Schizophrenia Research*, *161*(2–3), 386–391.
6. Hasson-Ohayon, I., Goldzweig, G., Lavi-Rotenberg, A., Luther, L., & Lysaker, P. H. (2018). The centrality of cognitive symptoms and metacognition within the interacting network of symptoms, neurocognition, social cognition and metacognition in schizophrenia. *Schizophrenia Research*, *202*, 260–266.
7. James, A. V., Johannesen, J. K., & Lysaker, P. H. (2018). Neurocognitive and metacognitive profiles of intact social cognition in prolonged schizophrenia. *The Journal of Nervous and Mental Disease*, *206*(12), 907–912.
8. Kolavarambath, R., Sudhir, P. M., Prathyusha, P. V., & Thirthalli, J. (2020). Emotion recognition, emotion awareness, metacognition, and social functioning in persons with schizophrenia. *Indian Journal of Psychological Medicine*, *42*(2), 147–154.
9. Lepage, M., Buchy, L., Bodnar, M., Bertrand, M.-C., Joober, R., & Malla, A. (2008). Cognitive insight and verbal memory in first episode of psychosis. *European Psychiatry*, *23*(5), 368–374.
10. Luther, L., Firmin, R. L., Vohs, J. L., Buck, K. D., Rand, K. L., & Lysaker, P. H. (2016). Intrinsic motivation as a mediator between metacognition deficits and impaired functioning in psychosis. *British Journal of Clinical Psychology*, *55*(3), 332–347.
11. Lysaker, P. H., Chernov, N., Moiseeva, T., Sozinova, M., Dmitryeva, N., Alyoshin, V., Kukla, M., Wiesepape, C., Karpenko, O., & Kostyuk, G. (2021). The association of metacognition with emotion recognition and perspective taking in a Russian sample with psychosis. *Journal of Clinical Psychology*, *77*(4), 1034–1044.
12. Lysaker, P. H., Dimaggio, G., Buck, K. D., Callaway, S. S., Salvatore, G., Carcione, A., Nicolò, G., & Stanghellini, G. (2011). Poor insight in schizophrenia: Links between different forms of metacognition with awareness of symptoms, treatment need, and consequences of illness. *Comprehensive Psychiatry*, *52*(3), 253–260.
13. Lysaker, P. H., Dimaggio, G., Daroyanni, P., Buck, K. D., LaRocco, V. A., Carcione, A., & Nicolò, G. (2010). Assessing metacognition in schizophrenia with the Metacognition Assessment Scale: Associations with the Social Cognition and Object Relations Scale. *Psychology and Psychotherapy: Theory, Research and Practice*, *83*(3), 303–315.
14. Lysaker, P. H., Leonhardt, B. L., Brüne, M., Buck, K. D., James, A., Vohs, J., Francis, M., Hamm, J. A., Salvatore, G., & Ringer, J. M. (2014). Capacities for theory of mind, metacognition, and neurocognitive function are independently related to emotional recognition in schizophrenia. *Psychiatry Research*, *219*(1), 79–85.
15. Lysaker, P. H., Olesek, K. L., Warman, D. M., Martin, J. M., Salzman, A. K., Nicolò, G., Salvatore, G., & Dimaggio, G. (2011). Metacognition in schizophrenia: Correlates and stability of deficits in theory of mind and self-reflectivity. *Psychiatry Research*, *190*(1), 18–22.
16. Vohs, J. L., Lysaker, P. H., Liffick, E., Francis, M. M., Leonhardt, B. L., James, A., Buck, K. D., Hamm, J. A., Minor, K. S., & Mehdiyoun, N. (2015). Metacognitive capacity as a predictor of insight in first-episode psychosis. *The Journal of Nervous and Mental Disease*, *203*(5), 372–378.
17. Zhang, Q., Li, X., Parker, G. J., Hong, X., Wang, Y., Lui, S. S., Neumann, D. L., Cheung, E. F., Shum, D. H., & Chan, R. C. (2016). Theory of mind correlates with clinical insight but not cognitive insight in patients with schizophrenia. *Psychiatry Research*, *237*, 188–195.

***Supplementary Material S1***

Complementary results.

Five studies employed regression analyses. Of these, two studies observed that the MAS-A subscale scores predicted the BLERT scores (15,71), one study reported that the MAS-M predicted the SCORS-USC scores (70). Aydin et al. (69) observed that the RMET predicted the MAS-A total score, the MAS-SR, and the MAS-O, but not the other subscales. Hamm et al. (86) found that the MAS-A total score and the BLERT were not only correlated at baseline but also six months after; and that the BLERT score significantly predicted the MAS-A total score at six months.

Furthermore, James et al. (71) observed a difference in the MAS-A score between participants with the BLERT scores below cut-off and those without, but no differences between those with or without the SAT-MC scores below cut-off.

Finally, two studies included control groups. One study identified a correlation between the Faux-Pas Task and the MAS-O scores, as well as between the FEIT and the MAS-D scores in healthy subjects, but not with other MAS-A subscales scores (21). In the other study, which included participants with a substance abuse disorder as a control group, no significant correlation was found between the MAS-A and the BLERT, but a correlation was identified between the MAS-D and the MAS-O subscales (15).

***Supplementary Material S2***

Risk of bias analysis.

Individual risks of bias in the included reports are presented in Table 10. Among the eight reports that were rated as having medium medication bias, one was rated as medium because of a homogeneous medication (49), and seven included patients with stable medication 30 days prior to inclusion. Blinding of metacognition ratings was not described in five reports, although three reports used the self-rated questionnaire BCIS as a metacognition assessment (20,73,74). Concerning the validation of the assessments, four reports that were rated as low used a rewritten version of the Hinting Test in American English to provide a translation that was culturally adapted for their population. Several studies used a translated version of the MAS in Turkish (69), Russian (46) or Indian (48). Bonfils et al. used an English version of the Derntl task (68). Zhang et al. used a translated version of the BCIS (20). Finally, five reports had a high risk of bias for the validation of the assessment because they did not specify if they used a translated version of the BCIS (48), the MAS and FEIT (21), the Ekman 60 Faces Test (46), the Yoni Task (20) or the Picture Sequencing Task (47). One report reported the number of participants in each step of the inclusion procedure (48). Two reports mentioned nonexploitable data due to neuropsychological performances (13,73), and one report excluded outliers in terms of biological data (69). Among the seven reports rated as medium for the outcome criterion, one used correlations between metacognition and social cognition as preliminary analyses (75). The six others used these correlations to address their secondary objective. Other potential biases were identified in the included studies. First, some reports did not specify the coefficient values for the correlation between social cognition scores and MAS scores (13,48,49) or BCIS scores (49). Furthermore, one report assessed social cognition and metacognition in two different clinical trials that were conducted asynchronously, with an interval of up to seven months (70).

**Table S1**

*Classification table for the risk of bias*

| **Risk of bias** | **Low** | **Medium** | **High** |
| --- | --- | --- | --- |
| Medication | Homogeneous and stable medication | Homogeneous or stable medication | Heterogeneous and not stable medication or unspecified |
| Financial conflict of interest | Absence of conflict of interest | Unspecified | Presence of conflict of interest |
| Financial compensation | Absence of compensation | Unspecified | Presence of compensation |
| Blindness of metacognition rating | Independent rating | / | Rating by the same assessor |
| Validation of the assessments | Validation in the language of the participants | Translation in the language of the participants | No validation or translation |
| Multiple testing correction | One analysis or several analyses with a correction | / | Several analyses without a multiple testing correction |
| Missing or incomplete data | Number of participants or data reported for each step of the procedure | Number of missing/incomplete data specified | Missing/incomplete data non specified |
| Ethical committee approval | Approval by a committee | / | No ethical committee |
| Primary or secondary outcome | Primary outcome | Secondary outcome | Ancillary study |

**Table S2**

*Characteristics and results of the reports included in the meta-analysis and review (n = 12) or only the review (n = 5)*

| **Authors** | **Design** | **Subjects** | **Social cognition assessment** | **Metacognition assessment** | **Results (Pearson’s *r*)** |
| --- | --- | --- | --- | --- | --- |
| **Meta-analysis and review** | | | | | |
| Aydın et al., 2018 | CC | 34 SCZ (31 HV) | RMET | MAS-A | RMET ***COR*** MAS-A (.43*) |
| Bonfils, Haas, et al., 2020 | CS | 57 SSD | Derntl tasks | MAS-A | Happiness ***COR*** MAS-SR (.31*) and MAS-D (.29*)  Happiness ***NO COR*** MAS-O (.15) and MAS-M (.17)  Neutral ***COR*** MAS-SR (.45**) and MAS-D (.32*)  Neutral ***NO COR*** MAS-O (.23) and MAS-M (.21)  Anger ***COR*** MAS-SR (.26*)  Anger ***NO COR*** MAS-M (.24), MAS-O (.08) and MAS-D (.06)  Disgust ***COR*** MAS-SR (.45**), MAS-D (.33*), MAS-M (.33*) and MAS-O (.29*)  Sadness ***COR*** MAS-SR (.33*) and MAS-O (.33*)  Sadness ***NO COR*** MAS-M (.24) and MAS-D (.22)  Fear ***COR*** MAS-SR (.28*) and MAS-O (.28*)  Fear ***NO COR*** MAS-D (.20) and MAS-M (.19) |
| Hamm et al., 2012 | P | 49 SSD | BLERT | MAS-A | BLERT ***COR*** MAS-A (.46**) |
| Hasson-Ohayon et al., 2015 | CC | 39 SCZ (60 HV) | Faux-Pas, FEIT | MAS-A | Faux-pas ***COR*** MAS-SR (.43**) and MAS-O (.29*)  Faux-pas ***NO COR*** MAS-M (.23) and MAS-D (.26)  FEIT ***COR*** MAS-SR (.36*), MAS-O (.38**), MAS-M (.32*) and MAS-D (.27*) |
| Hasson-Ohayon et al., 2018 | CS | 81 SSD | BLERT, Hinting Test, MCCB-SC, Picture-sequencing Task, SAT-MC | MAS-A | MCCB-SC ***COR*** MAS-SR (.30**) and MAS-M (.26*)  MCCB-SC ***NO COR*** MAS-O (.17) and MAS-D (.18)  Picture-sequencing Task ***COR*** MAS-SR (.32**), MAS-O (.27*) and MAS-D (.30**)  Picture-sequencing Task ***NO COR*** MAS-M (.09)  BLERT ***COR*** MAS-SR (.32**), MAS-O (.25*) and MAS-M (.23*)  BLERT ***NO COR*** MAS-D (.20)  Hinting Test ***COR*** MAS-SR (.27*)  Hinting Test ***NO COR*** MAS-O (.13), MAS-D (.09) and MAS-M (.15)  SAT-MC ***COR*** MAS-D (.24*)  SAT-MC ***NO COR*** MAS-SR (.23), MAS-O (.22) and MAS-M (.10) |
| James et al., 2018 | CS | 72 SSD | BLERT, Hinting Test, MCCB-SC, SAT-MC | MAS-A | MAS-A ***COR*** BLERT (.31**) and MCCB-SC (.30*)  MAS-A ***NO COR*** Hinting Test (.19) and SAT-MC (.22) |
| Lepage et al., 2008 | CS | 51 FEP | MCCB-SC | BCIS | MCCB-SC ***NO COR*** BCIS-SR (.20) and BCIS-SC (.22) |
| Luther et al., 2016 | CS | 175 SSD | BLERT | MAS-A | BLERT ***COR*** MAS-A (.33**) |
| Lysaker et al., 2010 | CS | 37 SSD | SCORS-USC | MAS-A | SCORS-USC ***COR*** MAS-A (.42**) |
| Lysaker et al., 2014 | CC | 115 SSD (58 Substance Use) | BLERT, Hinting Test, RMET | MAS-A | MAS-A ***COR*** BLERT (.53***), RMET (.26**) and Hinting Test (.26**) |
| Lysaker, Olesek, et al., 2011 | P | 36 SSD | BLERT, Hinting Test, RMET | BCIS | BCIS-SR ***NO COR*** RMET (-.10), Hinting Test (.16) and BLERT (.07)  BCIS-SC ***NO COR*** RMET (.10), Hinting Test (.15) and BLERT (.01) |
| Lysaker et al., 2021 | CC | 37 SSD, 41 SCZ (30 Major dépression) | Ekman 60 Faces Test | MAS-A | SSD : Ekman 60 Faces Test ***COR*** MAS-A (-.46**) |
|  |  |  |  |  | SCZ : Ekman 60 Faces Test ***COR*** MAS-A (.57**) |
| **Review** | | | | | |
| Dubreucq et al., 2022 | CC | 26 SSD (30 Autism spectrum disorder) | MASC | MAS-SR | MASC ***NO COR*** MAS-SR (.20) |
| Kolavarambath et al., 2020 | CC | 27 SSD (26 HV) | TRENDS | MAS-A, BCIS-SR | TRENDS ***NO COR*** BCIS-SR (-.10), MAS-SR (.21) and MAS-O (.23) |
| Lysaker, Dimaggio, et al., 2011 | CS | 65 SSD | Hinting Test | MAS-A | Hinting test ***NO COR*** MAS-A |
| Vohs et al., 2015 | CS | 40 FEP | BLERT, Hinting Test | MAS-A, BCIS-SR | BLERT ***COR*** MAS-A (.40*) and MAS-O (.38*) |
| Zhang et al., 2016 | CC | 56 SCZ (30 HV) | Yoni Task | BCIS | BCIS composite score ***NO COR*** Yoni task Affective first order (.06) and Affective second order (.07)  BCIS composite score ***NO COR*** Yoni task Cognitive first order (.01) and Cognitive second order (.21) |

*Note.* CC = Case-Control; CS = Cross-Sectional; P = Prospective; SCZ = Schizophrenia; HV = Healthy Volunteers; SSD = Schizophrenia Spectrum Disorders; FEP = First Episode Psychosis; RMET = Reading the Mind in the Eyes Test; BLERT = Bell Lysaker Emotion Recognition Task; FEIT = Face Emotion Identification Task; SAT-MC = Social Attribution Task - Multiple Choice; MCCB-SC = Matrics Consensus Cognitive Battery - Social Cognition; SCORS-USC = Social Cognition and Object Relations Scale - Understanding Social Causality; TRENDS = Tool for Recognition of Emotions in Neuropsychiatric DisorderS; MASC = Movie for the Assessment of Social Cognition; MAS-A = Metacognitive Assessment Scale - Abbreviated; BCIS = Beck Cognitive Insight Scale; BCIS-SR = BCIS - Self-Reflectiveness scale; BCIS-SC = BCIS - Self-Certainty scale; MAS-SR = MAS - Self-Reflectivity subscale; MAS-D = MAS - Decentration subscale; MAS-O = MAS - understanding Other’s mind subscale; MAS-M = MAS - Mastery subscale. COR = significant correlation; NO COR = non-significant correlation.

*p<.05, **p<.01, ***p<.001.

**Table S3**

*Subgroup analysis comparing studies using the BLERT and other emotion processing assessments*

| **Assessment** | ***r*** | **95% CI** | ***p*** | ***I2*** | ***p***  **subgroups** |
| --- | --- | --- | --- | --- | --- |
| BLERT | .35 | .17, .50 | .05 | 54.85% | .42 |
| Other | .19 | -.52, .75 | <.001 | 88.06% |  |

**Table S4**

*Risks of bias of the reports included in the review (n=17)*

| **Authors** | **Medica-tion** | **Financial conflict of interest** | **Financial compensa- tion** | **Blindness of metacognition rating** | **Validation of the assessment** | **Multiple testing correction** | **Missing or incomplete data** | **Ethical committee approval** | **Primary or Secondary outcome** |
| --- | --- | --- | --- | --- | --- | --- | --- | --- | --- |
| Aydın et al., 2018 | Low | Medium | Medium | Low | Medium | High | Medium | Low | Medium |
| Bonfils, Haas, et al., 2020 | High | Low | High | High | Medium | High | High | Low | High |
| Dubreucq et al., 2022 | High | Low | Medium | Hight | Low | High | High | Low | Medium |
| Hamm et al., 2012 | Medium | Medium | Medium | Low | Low | High | High | Low | Low |
| Hasson-Ohayon et al., 2015 | High | Low | Medium | Low | High | High | High | Low | High |
| Hasson-Ohayon et al., 2018 | Medium | Low | Medium | Low | High | High | High | Low | High |
| James et al., 2018 | Medium | Low | Medium | Low | Low | High | High | Low | High |
| Lepage et al., 2008 | High | Medium | Medium | High | Low | High | Medium | High | Low |
| Luther et al., 2016 | High | Medium | Medium | Low | Low | High | High | High | Medium |
| Lysaker et al., 2010 | Low | Medium | Medium | Low | Low | Low | High | Low | High |
| Lysaker et al., 2014 | Medium | Medium | Medium | Low | Low | High | High | High | Medium |
| Lysaker, Olesek, et al., 2011 | Medium | Medium | Medium | High | Low | High | High | High | Medium |
| Lysaker et al., 2021 | High | Medium | Medium | Low | High | High | High | Low | Low |
| Vohs et al., 2015 | Medium | Low | Medium | Low | Low | High | High | Low | Medium |
| Lysaker, Dimaggio, et al., 2021 | Medium | Medium | Medium | Low | Low | High | Medium | High | High |
| Zhang et al., 2016 | High | Low | Medium | High | High | High | High | Low | Medium |
| Kolavarambath et al., 2020 | Medium | Low | Low | High | High | High | Low | Low | Medium |

**Table S5**

*Analysis of publication bias by social cognition function.* *p<.05

| **Social cognitive functions** | **Egger regression** | **Begg and Mazumdar** |
| --- | --- | --- |
| Theory of Mind | t = 0.11, p = .92 | z = -0.49, p = .62 |
| Affect recognition | t = -1.28, p = .24 | z = -1.16, p = .25 |
| Attribution | t = 26.78, **p =.02*** | z = 1.57, p = .12 |
| Social cognition - other | t = 0.34, p = .76 | z = 0.00, p = 1.00 |
